# Promotion of Orange-Fleshed Sweet Potato Increased Vitamin A Intakes and Reduced the Odds of Low Retinol-Binding Protein among Postpartum Kenyan Women^1^,^2^,^3^

**DOI:** 10.3945/jn.116.236406

**Published:** 2017-04-12

**Authors:** Amy Webb Girard, Frederick Grant, Michelle Watkinson, Haile Selassie Okuku, Rose Wanjala, Donald Cole, Carol Levin, Jan Low

**Affiliations:** 4 Hubert Department of Global Health, Rollins School of Public Health, Emory University, Atlanta, GA;; 5 International Potato Center, Nairobi, Kenya;; 6 Division of Epidemiology, Dalla Lana School of Public Health, University of Toronto, Toronto, Ontario, Canada; and; 7 Department of Global Health, University of Washington, Seattle, WA

**Keywords:** agriculture, maternal nutrition, vitamin A, orange-fleshed sweet potato, Kenya

## Abstract

**Background:** Orange-fleshed sweet potato (OFSP) improves vitamin A (VA) status of young children; research with pregnant and lactating women is limited.

**Objective:** We examined the effectiveness of the Mama SASHA (Sweetpotato Action for Security and Health in Africa) program to improve nutrition knowledge, diets, and nutritional status of pregnant and lactating women (PLW) in Western Kenya.

**Methods:** Eight health facilities were allocated to the Mama SASHA intervention or comparison arms. PLW in intervention facilities received enhanced nutrition counseling at health clinics, were linked with community-based maternal support groups, and received vouchers for OFSP vine cuttings. Control PLW received clinic-based nutrition counseling only. A total of 505 women in early and midpregnancy, attending their first antenatal care visit, and with no previous engagement in project activities were enrolled from the 8 facilities. Nutrition and health-seeking knowledge, food security, dietary patterns, and anthropometric measurements were collected at 4 time points at ≤9 mo postpartum. VA intakes were assessed with multipass 24-h recalls in a subsample of 206 mothers at 8–10 mo postpartum. VA status was assessed by using serum retinol-binding protein (RBP). Impacts were estimated with multilevel mixed models adjusted for clustering and differences at enrollment.

**Results:** At enrollment, 22.9% of women had RBP <1.17 μmol/L. By 9 mo postpartum, intervention women had significantly higher intakes of VA [adjusted difference = 297.0 retinol activity equivalent (RAE) units; 95% CI: 82, 513 RAE units; *P* = 0.01; *n* = 206], greater consumption of VA-rich fruit and vegetables in the previous 7 d (difference-in-difference estimate: 0.40 d; 95% CI: 0.23, 0.56 d; *P* < 0.01), and a 45% reduction in the odds of RBP <1.17 μmol/L (OR: 0.55; 95% CI: 0.33, 0.92; *P* = 0.01).

**Conclusion:** Promotion of OFSP to PLW through health services is a feasible strategy to improve women’s nutrition knowledge, VA intakes, and maternal RBP.

## Introduction

Orange-fleshed sweet potato (OFSP)^8^, as a staple food, supplies substantial vitamin A (VA) and energy. Over the past decade, strong evidence has shown the potential of OFSP to improve VA intakes of women and children and child VA status (1–4). However, research on the effectiveness of OFSP to improve VA status of pregnant and lactating women (PLW) is limited. This oversight is despite the realization that this group is both *1*) vulnerable to VA deficiency due to increased physiologic needs and *2*) is a group whose status is critical for infant adequacy due to VA transfer in utero and through breast milk.

The Sweetpotato Action for Security and Health in Africa (Mama SASHA) project, a 5-y cluster-allocated proof-of-concept project, explicitly integrated agriculture and nutrition interventions into antenatal care (ANC) and postnatal care (PNC) health care services in Western Kenya. Through community and health facility platforms, the project targeted households with PLW with enhanced nutrition education by using inputs in the form of vouchers for OFSP planting materials and enhanced agricultural extension (5). Demographic and Health Survey data available just before program development indicated undernutrition and low coverage with VA supplementation programs as public health concerns for young children and women of reproductive age in Western Kenya (6). Data on maternal VA status were not available at that time; however, a 2003 study in the neighboring Nandi district noted that, among 88 postpartum women, 78.1% had low breast-milk retinol concentrations and 10% had severe VA deficiency on the basis of serum retinol <0.35 μmol/L (7). Referencing the project’s impact pathway (5), we hypothesized that Mama SASHA activities would improve the nutritional status of PLW through increased uptake of health services and enhanced production and consumption of OFSP.

## Methods

### 

#### Study site and program activities.

Mama SASHA was implemented in 2 counties in Western Kenya. A full description of the program, including its development and design, activities, and monitoring and evaluation strategy, is presented in Cole et al. (5). Briefly, at the start of program activities in 2010, 8 health facilities were purposively selected according to size-related variables, location criteria, and coverage with community health workers (CHWs) linked to the APHIA II/APHIAplus (AIDS, Population and Health Integrated Assistance) program. The APHIA II/APHIAplus program is a joint health capacity–strengthening program implemented by the US Agency for International Development and PATH (formerly Program for Appropriate Technologies in Health). The selected health facilities were randomly allocated to intervention or control. Each facility served 3–4 sublocations with 10–15 villages/sublocation. Facilities were selected such that the distance between them was ∼30 and 50 km apart to reduce contamination.

As part of the APHIAplus program, CHWs in all communities mobilized women to attend ANC and PNC. In intervention facilities, women attending ANC or early PNC clinics were provided with enhanced nutrition education, which included, in addition to standard-of-care counseling for maternal nutrition and infant and young child feeding (IYCF) practices, additional information on VA, OFSP as a VA-rich food, and the importance of VA for maternal and child health. At ANC visits, women also received 2 vouchers (1 time/trimester and at the first postpartum visit), which they redeemed for 100 cuttings each of varieties of OFSP. Kabode (CIP and National Crops Resources Research Institute Uganda; NASPOT-10-O/SPK004/6/6) and Vita (CIP and National Crops Resources Research Institute Uganda; NASPOT-9-O/SPK004/6) varieties. Kabode and Vita contain 9655 and 7460 mg β-carotene/100 g raw, fresh weight, respectively. Women redeemed vouchers for vine cuttings from secondary vine multipliers located near the health clinics. The project selected and trained vine multipliers to produce clean planting materials, provide the women with vines, and deliver OFSP production support. In addition, trained agriculture extension officers followed up with the women to deliver agronomic advice and to assess and discuss OFSP planting and crop management. Last, women in intervention communities were linked with community-based PLW clubs where CHWs conducted monthly dialog sessions on nutrition and health, including the preparation of VA-rich foods and OFSP. Educational materials, including wall and flip charts and counseling cards, were developed by the Mama SASHA program for use by health workers during clinic-based counseling and mothers’ club sessions. At control sites, nurses and CHWs received training on nutrition and optimal IYCF counseling from the APHIAplus program; pregnant women’s clubs were not set up, and there were no agricultural or OFSP activities.

#### Longitudinal study design and population.

As part of the larger evaluation strategy (5), we designed and implemented a longitudinal cohort study that followed women from their first ANC visit to 9 mo postpartum. From November 2012 to February 2013, trained health workers prescreened 3025 women attending the 8 facilities for eligibility criteria. Prescreening criteria included age 17–40 y, attending the first ANC visit, and being in early and midpregnancy (10–26 wk). Of those prescreened, 2307 were not eligible. Additional criteria were then applied and included planning to breastfeed and planning to live in their current village until 10 mo postpartum. Exclusion criteria included involvement in Mama SASHA activities during a previous pregnancy, living in an intervention village but visiting a control facility, or vice versa, or living outside of the Mama SASHA project catchment area (i.e., outside control and intervention villages). After prescreening, of the 718 eligible participants, 505 women were eligible upon additional screening and were consented and enrolled (control = 255, intervention = 250).

#### Data collection.

Trained research assistants administered an extensive questionnaire at enrollment and at 3 subsequent visits in late pregnancy at 4 and at 9 mo postpartum. Questionnaires collected detailed information on sociodemographic characteristics, household food security and diet diversity, maternal and infant diet and consumption of VA-rich foods, maternal health and nutrition knowledge, sweet potato production, and participation in program activities. Maternal weight and maternal midupper arm circumference (MUAC) were measured in duplicate by using a Seca 874 scale and flexible, nonstretchable tape, respectively, at each visit. Questionnaire and anthropometric data were double-entered into Census and Survey Processing System Software (CSPro), version 6.0 (US Census Bureau and Inner-City Fund International). On the day of data collection by 2 separate research assistants.

Research assistants obtained finger-prick capillary blood samples between 0800 and 1600 by using single-use sterile Contact-Activated BD Microtainer Lancets (Becton, Dickinson and Company). Blood was collected in BD Microtainer tubes with EDTA (Becton, Dickinson and Company). Hemoglobin was determined within 3 min of blood collection with the use of the Hemocue 201+ system (Radiometer Medical Aps) machine and adjusted for altitude (8). An additional 400–500 mL capillary blood was collected into BD Microtainer tubes with EDTA and, within 12 h of blood collection, centrifuged at 1500 × *g* for 5 min at 27°C. Plasma was then removed and stored in Nalgene cryogenic tubes (Thermo Fisher Scientific) at −20°C. Samples were transported on dry ice to Germany for subsequent laboratory analysis of retinol-binding protein (RBP), C-reactive protein (CRP), and α1-acid glycoprotein (AGP) by using a sandwich ELISA technique (9, 10). All of the samples were measured in duplicate and the intra- and interassay CVs were <10%. A summary table of data collection and timing is available in the online supplemental material (**Supplemental Table 1**).

#### Sample size estimation.

From the 2010 baseline household survey data on predictors of child VA status conducted in Mama SASHA control and intervention communities, we estimated the intraclass correlation coefficient for infant VA status to be 0.02 (11, 12). Data on RBP in women were not available for this population. However, assuming a population SD in breast-milk retinol of 0.7 μmol/L (7), we estimated that 400 women would be required to detect a significant double difference in breast-milk retinol of 0.1 μmol/L, with 95% confidence and 80% power (13). Recruiting an additional 25% for anticipated losses to follow-up yielded an estimated required sample size of 500 women.

#### Multipass 24-h recalls.

From December 2013 to February 2014, a subsample of 206 women enrolled in the cohort study and who had reached 8–10 mo postpartum completed interactive, multipass 24-h dietary recalls (14), with a repeat recall conducted on a separate day for a subset of 79 mother-child pairs (*n* = 44 control, *n* = 35 intervention). Standard recipes were developed for mixed dishes that were frequently consumed and prepared in a similar way in the study area. We adopted nutrient values from the HarvestPlus Food Composition Table for East and Central Uganda (15) with modifications to reflect Kenyan context and revising the β-carotene and VA values for the OFSP varieties distributed in the Mama SASHA project. The retention of β-carotene after cooking was accounted for (1, 2, 16). Because the intervention dictated that vines were distributed in a 1:1 ratio, we assumed that half of all OFSP consumed was Vita and the other half was Kabode. Because most foods and preparation methods in Western Kenya are similar to those in Eastern Uganda, and similar measurement methods were used to estimate food intake, a near-comprehensive conversion factor database was developed on the basis of Ugandan values, with some modifications made for the following: *1*) the size of local avocadoes and tomatoes, *2*) the maize-based stiff porridge *Ugali*, and *3*) *Mushenye,* a new dish specific to Kenya. Collection of new volume-to-weight conversions for standard recipes was performed. All of the dietary data were double-entered, verified, and processed in CS Dietary (Serpro).

#### Ethics.

All research protocols were approved by the institutional review boards at Emory University and the Kenya Medical Research Institute. All of the participants provided written informed consent before participation and were orally re-consented at each study visit.

#### Variable specification.

A wealth index was created from the assets and household characteristic variables collected at enrollment (11, 17). At each visit, food insecurity in the previous 30 d and household dietary diversity in the previous 24 h were assessed by using the FANTA (Food and Nutrition Technical Assistance III) Household Food Insecurity Access Scale (18) and the Household Dietary Diversity Scale (19), respectively. Women’s dietary diversity scores (WDDSs) were estimated from a 7-d FFQ of 58 foods (20), which were then categorized by using the updated categories described by the FAO (21). Because data were collected as the number of days a specific food was consumed in the previous 7 d as opposed to consumption of the food in the previous 24 h, we estimated WDDSs on the basis of whether a food group was consumed on 3 of the previous 7 d. Maternal knowledge scores, collected at enrollment and the fourth visit, were estimated by summing the number of correct responses to a series of 10 questions on IYCF, nutrition and VA, and health-seeking knowledge and attitudes for a total possible score of 14 points. The change in nutrition scores from enrollment to visit 4 was estimated and used as the outcome variable.

RBP concentrations were corrected for inflammation by using the Thurnham correction factor method (22), where elevated CRP was defined as >5 mg/L and elevated AGP as >1 g/L. Low RBP status was defined as RBP <1.17 μmol/L on the basis of cutoffs derived from a nationally representative survey in PLW in Cameroon with similar a background prevalence of inflammation (23, 24). Anemia was defined as altitude-adjusted hemoglobin <110 g/L in pregnancy and 120 g/L in the postpartum period (8). We did not adjust hemoglobin for inflammation because we noted no consistent, significant correlations between markers of inflammation and hemoglobin concentrations (**Supplemental Table 2**).

#### Statistical analysis.

With the use of cluster-adjusted regression analysis we examined differences between intervention and control groups at enrollment for sociodemographic characteristics, food security, and dietary diversity among those included in 24-h recalls compared with those not included. We similarly assessed bias due to loss to follow-up by comparing mothers completing the fourth visit and those lost to follow-up.

For intake data from 24-h recalls, macronutrients were adjusted per 100 kcal and micronutrients were adjusted per 1000 kcal. When 2 recalls were available, the mean intake was estimated; otherwise, the estimated intake was based on the single recall. Intake data were nonnormally distributed; we therefore applied Box-Cox transformation before modeling and estimated effects by using both the nontransformed and the transformed energy-adjusted intakes. VA intakes were modeled as continuous outcomes and as the odds of intake adequacy with adequacy evaluated by using 2 cutoffs; the DRI for the recommended daily intake of VA [1300 μg retinol activity equivalents (RAEs)/d for lactating women] and the daily Estimated Average Requirement for VA for lactating women (800 μg RAEs/d) (25, 26).

Linear mixed models were used to assess intervention effects on continuous outcomes specifying random intercept, random slopes, and unstructured covariance structures (27, 28). Restricted maximum likelihood estimation was used to generate robust SEs and confidence limits. Difference in difference (DiD) estimates were estimated from a treatment group and visit number (time) interaction term in longitudinal models adjusted for clustering and fixed-effects covariates. Linear mixed models were also used to estimate, by visit number, least-squares means and SEs for continuous outcomes and intervention effects adjusted for clustering, fixed-effects covariates, and values at enrollment. Generalized linear mixed models for outcomes where the measure was a dichotomous variable were estimated specifying a binomial distribution, link = logit function, and unstructured covariance structures (29). Intraclass correlation coefficients and variance estimates suggested that the contributions of cluster-level (sublocation) random effects to the variance for most outcomes with repeated measures were near zero and the inclusion of cluster random effects worsened the model fit. Hence, this level of clustering was dropped from longitudinal analysis; exceptions included MUAC and WDDS. Sublocation random effects were also retained for analyses of VA intakes, change in knowledge scores, and all analyses stratified by visit number. Variables that differed significantly between intervention and control groups at enrollment and potentially influential fixed-effects covariates identified a priori from the literature were evaluated for inclusion in final models by using percentage change in the effect estimate and model fit criteria.

## Results

### Enrollment characteristics, loss to follow-up, and project participation

Women enrolled were 24.3 ± 5.5 y old and the majority had completed primary education (**Table 1**). For 30.3% of women this was their first pregnancy. In cluster-adjusted analyses, significant differences between intervention and control groups existed at enrollment for sociodemographic characteristics related to who served as head of household, maternal education, household head education, and household dietary scores (Table 1). Of the 505 women (250 intervention, 255 control) enrolled, 383 women (178 intervention, 205 control) had data available at visit 2 in late pregnancy, 401 (196 intervention, 205 control) at visit 3 at 4 mo postpartum, and 385 (193 intervention, 192 control) at visit 4 at 9 mo postpartum. Over the course of the follow-up period, 60 participants were excluded from further follow-up because of fetal (*n* = 26), infant (*n* = 20), or maternal (*n* = 1) death; severe illness (*n* = 1); preterm delivery (*n* = 4); and multiple births (*n* = 8). Forty-three relocated permanently outside of the study sites. Travel, especially around the time of delivery, and refusal to participate in a particular visit contributed to missing visits. Exclusions and loss to follow-up did not differ by treatment group. However, it should be noted that the proportion who did not attend the second visit was significantly higher for the intervention group (29%) than for the control group (20%). Overall, mothers who were lost to follow-up were more likely to have been divorced at enrollment, but no other differences were observed. The subsample of 206 mother and infant pairs (102 control, 104 intervention) who participated in 24-h recalls at 8–10 mo postpartum were similar to those who did not participate with respect to sociodemographic characteristics and dietary patterns at enrollment, with the exception of having greater household dietary diversity adequacy at enrollment (*P-*difference = 0.04; **Supplemental Table 3**).

**TABLE 1:** Enrollment and birth characteristics of the 505 women participating in the Mama SASHA cohort study in Western Kenya^1^

	Overall	INT	CON	*P*-difference^2^
Descriptive characteristics				
* n*	505	251	254	
Maternal age, y	24.3 ± 5.5	24.1 ± 5.5	24.6 ± 5.5	0.27
Primiparous, *n* (%)	153 (30.3)	79 (31.6)	74 (29.0)	0.47
Gestational age at enrollment, wk	20.4 ± 5.1	20.5 ± 5.5	20.4 ± 4.7	0.72
Mother is married/partnered, monogamous, *n* (%)	399 (79.0)	194 (77.6)	205 (80.4)	0.41
Head of household is husband/partner, *n* (%)	432 (85.5)	205 (82.0)	227 (89.0)	0.03
Maternal education, *n* (%)				0.02
<8 y	155 (30.7)	87 (34.1)	68 (27.2)	
Completed 8 y (primary)	157 (31.1)	79 (31.0)	78 (31.2)	
>8–12 y	162 (32.1)	85 (34.0)	77 (30.2)	
>12 y	31 (6.1)	12 (4.7)	19 (7.6)	
Maternal occupation, *n* (%)				0.38
Does not work remuneratively	200 (39.8)	120 (48.2)	80 (31.4)	
Agriculture	168 (33.4)	63 (25.3)	105 (41.2)	
Salaried employment	25 (5.0)	15 (6.0)	10 (3.9)	
Other	110 (21.9)	59 (23.2)	51 (20.5)	
Head of household education, *n* (%)				0.001
<8 y	99 (19.6)	49 (19.6)	50 (19.6)	
Completed 8 y (primary)	122 (24.2)	45 (18.0)	77 (30.2)	
>8–12 y	189 (37.4)	91 (36.4)	98 (38.8)	
>12 y	95 (18.8)	65 (26.0)	30 (11.8)	
Head of household occupation, *n* (%)				0.13
Does not work	68 (13.5)	47 (18.10)	21 (8.2)	
Agriculture	104 (18.7)	31 (12.5)	63 (24.7)	
Salaried employment	81 (16.1)	44 (17.7)	37 (14.5)	
Casual laborer	116 (23.1)	56 (22.6)	60 (23.5)	
Informal business or other self-employment	144 (28.6)	70 (28.2)	74 (29.0)	
Wealth/asset index score	8.55 ± 1.77	8.54 ± 1.92	8.55 ± 1.62	0.89
Children <5 y of age,^3^ *n*	1 (0–2)	1 (0–1)	1 (0–2)	0.49
Household food insecurity category				0.93
Secure/mild	276 (55.1)	131 (54.1)	145 (57.8)	
Moderate	102 (20.6)	58 (24.0)	44 (17.5)	
Severe	115 (23.3)	53 (21.1)	62 (24.7)	
Household Dietary Diversity Scale score	5.45 ± 1.42	5.17 ± 1.36	5.73 ± 1.43	0.01
Birth outcomes				
*n*	402	197	205	
Gestational age at delivery, wk	39.6 ± 1.12	38.8 ± 3.6	39.2 ± 3.5	0.28
Infant weight within 1 wk of delivery, kg	3.42 ± 0.55	3.39 ± 1.03	3.37 ± 0.86	0.94
Infant sex is female, *n* (%)	187 (46.8)	94 (48.2)	94 (45.4)	0.57

1 Values are means ± SDs unless otherwise indicated. CON, control; INT, intervention; Mama SASHA, Sweetpotato Action for Security and Health in Africa.

2 *P*-difference estimated by using cluster-adjusted regression analyses.

3 Values are medians (IQRs).

Participation data indicated that 93% of intervention participants attending the fourth visit (*n* = 385) ever received vouchers for OFSP vines; the mean number of times received was 2.8 ± 1.2. However, only 23% of intervention women reported ever participating in community-based PLW’s clubs. Eight intervention women reported no participation in any Mama SASHA activities.

#### Impacts on nutrition knowledge.

At enrollment, participants did not differ significantly on total scores for nutrition and health knowledge (*P*-difference = 0.45) or the subdomain for IYCF knowledge (*P*-difference = 0.15). However, intervention women scored significantly higher on the subdomain related to VA knowledge at enrollment (*P-*difference = 0.04; **Table 2**). Intervention mothers showed a significantly greater 2.6- ± 2.7-point increase in overall nutrition and health knowledge scores from enrollment to 9 mo postpartum compared with a 1.6- ± 2.2-point increase among control mothers (*P-*DiD = 0.01). Scores on domains of VA knowledge increased by 1.0 ± 1.5 points in intervention mothers compared with 0.6 ± 1.2 points among control mothers (*P-*DiD < 0.001). Knowledge scores on IYCF domains increased similarly in both groups (Table 2).

**TABLE 2:** Effects of an integrated agriculture, nutrition, and health intervention on nutrition and health knowledge scores among a cohort of 505 women participating in the Mama SASHA cohort study in Western Kenya^1^

	Scores at enrollment			Mean increase in scores from enrollment to the 9-mo postpartum visit		
Index scores (possible range)	INT (*n* = 250)	CON (*n* = 255)	*P*-difference^2^	INT (*n* = 193)	CON (*n* = 192)	DiD (95% CI)^3^
Nutrition and health knowledge (0–14)	3.7 ± 2.3	3.4 ± 2.2	0.45	2.6 ± 2.7	1.6 ± 2.2	1.0 (0.1, 1.9)*
VA knowledge domain (0–4)	1.0 ± 1.2	0.6 ± 1.2	0.04	1.0 ± 1.5	0.2 ± 1.2	0.8 (0.4, 1.2)^‡^
IYCF knowledge domain (0–8)	2.2 ± 1.5	2.4 ± 1.3	0.15	1.5 ± 1.8	1.2 ± 1.4	0.4 (−0.3, 1.1)

1 Values are mean ± SDs. *P < 0.05, ^‡^*P* < 0.01. CON, control; DiD, difference in difference; INT, intervention; IYCF, infant and young child feeding; Mama SASHA, Sweetpotato Action for Security and Health in Africa; VA, vitamin A.

2 *P* values for the difference in mean scores at enrollment estimated from cluster-adjusted regression analyses.

3 Estimates and 95% CIs for differences in mean change in knowledge scores estimated from restricted maximum likelihood estimation by using mixed-effects models.

#### Impacts on OFSP consumption, dietary patterns, and VA intakes.

At enrollment, 21 (8.4%) intervention women consumed any OFSP in the previous 7 d compared with 1 woman from control facilities. The proportion of intervention women consuming any OFSP in the previous 7 d increased to 28.7% in late pregnancy and to 35.7% and 55.7% at the 4- and 9-mo postpartum visits, respectively (adjusted *P*-trend < 0.001). The number of control women consuming any OFSP in the previous 7 d did not exceed 1 woman at any time point. Because of the low proportion of women consuming OFSP in the control group across visits, impact estimates for the consumption of OFSP could not be estimated.

Neither the consumption of VA-rich fruit and vegetables nor WDDSs differed at enrollment (**Table 3**). At subsequent visits, the number of days consuming VA-rich fruit and vegetables and WDDSs were significantly higher among intervention women in adjusted models. In adjusted longitudinal analyses, the intervention significantly increased the number of days that VA-rich fruit and vegetables were consumed in the previous 7 d compared with the control by 0.4 d (adjusted *P*-DiD < 0.01), but the overall impact on WDDSs was nonsignificant.

**TABLE 3:** Effects of an integrated agriculture, nutrition, and health intervention on maternal dietary patterns, overall and by visit, for 505 women in Western Kenya enrolled in early to midpregnancy and followed through 9 mo postpartum^1^

	INT	CON	Difference (95% CI)
WDDS (possible range: 0–10)			
Visit 1 (enrollment)	5.5 ± 0.4	5.3 ± 0.4	0.2 (0.1, 0.5)*
Visit 2 (late third trimester)	5.4 ± 0.4	5.0 ± 0.4	0.4 (0.1, 0.7)**
Visit 3 (4 mo postpartum)	5.4 ± 0.40	5.2 ± 0.4	0.3 (0.0, 0.6)*
Visit 4 (9 mo postpartum)	6.1 ± 0.45	5.7 ± 0.4	0.4 (0.1, 0.7)**
Difference in difference^2^			0.1 (−0.1, 0.5)
Number of days consumed VA-FVs in past 7 d			
Visit 1 (enrollment)	4.6 ± 0.6	4.3 ± 0.6	0.3 (−0.1, 0.8)
Visit 2 (late third trimester)	2.7 ± 0.6	2.1 ± 0.7	0.6 (0.1, 1.1)^‡^
Visit 3 (4 mo postpartum)	2.7 ± 0.6	1.6 ± 0.7	1.0 (0.6, 1.5)^‡^
Visit 4 (9 mo postpartum)	2.5 ± 0.7	1.0 ± 0.7	1.5 (1.1, 2.0)^‡^
Difference in difference			0.4 (0.2, 0.6)^‡^

1 Values are cluster- and covariate-adjusted least-squares means ± SEs. A total of 505 women (250 INT, 255 CON) were enrolled and had data available at enrollment, 383 women (178 INT, 205 CON) had data available at visit 2, 401 (196 INT, 205 CON) at visit 3, and 385 (193 INT, 192 CON) at visit 4. *P < 0.10, **P < 0.05, ^‡^*P* < 0.01. CON, control; INT, intervention; VA-FV, vitamin A–rich fruit and vegetable; WDDS, women’s dietary diversity score (based on consuming food groups on 3 of 7 previous days).

2 Difference in difference estimates are the interaction term of treatment and time derived from restricted maximum likelihood estimations of longitudinal data by using mixed-effects models.

In the subsample of women participating in 24-h recalls, the intervention was associated with significantly higher intakes of β-carotene (*P*-difference = 0.01) and RAEs (*P*-difference = 0.01) at 8–10 mo postpartum (**Table 4**). Intervention women also had significantly higher odds of VA adequacy, measured as meeting either the DRI (*P* < 0.001) or the Estimated Average Requirement (*P* < 0.001; Table 4). OFSP ranked higher in percentage contributions to β-carotene and RAE intakes among intervention mothers than among control mothers (**Supplemental Table 4**).

**TABLE 4:** Effects of an integrated agriculture, nutrition, and health intervention on energy and energy-adjusted β-carotene and VA intakes and adequacy of VA intakes among 206 women at 8–10 mo postpartum participating in the Mama SASHA cohort study in Western Kenya^1^

	INT (*n* = 104)	CON (*n* = 102)	Adjusted difference (95% CI)^2^
β-Carotene, μg/d	1784 (992–3533)	1457 (927–2257)	145 (317, 2597) [0.01]
VA, μg RAEs/d	451 (251–810)	321 (173–553)	297 (82, 513) [0.01]
Energy, kcal/d	2586 (2122–3022)	2540 (2168–2894)	44 (−159, 246) [0.71]
Met DRI,^3^ *n* (%)	19 (18.3)	1 (1.0)	1.3 (1.2, 1.5)^¥^
Met EAR,^3^ *n* (%)	33 (31.7)	8 (7.8)	1.6 (1.3, 1.9)^¥^

1 Values are medians (IQRs) unless otherwise indicated. Micronutrients are presented as per 1000 kcal. ^¥^*P* < 0.001. CON, control; EAR, Estimated Average Requirement; INT, intervention; Mama SASHA, Sweetpotato Action for Security and Health in Africa; RAE, retinol activity equivalent; VA, vitamin A.

2 For ease of interpretation, estimates and 95% CIs were derived from models that used nontransformed intake data, whereas *P* values [shown in brackets] were estimated by using Box-Cox transformed data.

3 Based on the DRI and EAR for lactating women: DRI, 1300 μg RAEs/d; EAR, 900 μg RAE/d (25, 26).

### Maternal nutritional status

At enrollment, overall, 27.0% and 22.9% of women had uncorrected and inflammation-adjusted RBP values <1.17 μmol/L, respectively (**Table 5**; **Supplemental Tables 5** and **6**); 31% were anemic. Mean inflammation-adjusted RBP, hemoglobin, the proportion of those who were anemic, and the proportion with low RBP did not differ between groups at enrollment (Table 5). There were no significant impacts of the intervention on mean RBP concentrations or on hemoglobin over the follow-up period. Despite no overall impacts on mean hemoglobin or RBP, the intervention was associated with significantly reduced odds of anemia in late pregnancy (*P* < 0.001) and significantly reduced odds of RBP <1.17 μmol/L at 9 mo postpartum (*P* = 0.01).

**TABLE 5:** Effects of an integrated agriculture, nutrition, and health intervention on maternal RBP, hemoglobin, and nutritional status of 505 women participating in the Mama SASHA cohort study in Western Kenya^1^

	INT	CON	Adjusted difference (95% CI)^2^
RBP,^3^ μmol/L			
Visit 1 (enrollment)	1.52 ± 0.11	1.55 ± 0.11	−0.03 (−0.12, 0.06)
Visit 2 (late third trimester)	1.29 ± 0.08	1.26 ± 0.09	0.03 (−0.04, 0.10)
Visit 4 (9 mo postpartum)	1.53 ± 0.16	1.45 ± 0.17	0.09 (−0.03, 0.21)
Difference in difference			0.03 (−0.01, 0.07)
MUAC, cm			
Visit 1 (enrollment)	25.1 ± 0.9	26.6 ± 0.9	−1.5 (−2.2, −0.7)^‡^
Visit 2 (late third trimester)	26.1 ± 0.5	26.6 ± 0.6	−0.4 (−0.9, 0.0)
Visit 3 (4 mo postpartum)	25.6 ± 0.8	25.7 ± 0.9	−0.04 (−0.8, 0.7)
Visit 4 (9 mo postpartum)	25.7 ± 0.9	25.2 ± 1.0	0.5 (−0.3, 1.2)
Difference in difference			0.0 (−0.1, 0.4)
Hemoglobin,^4^ g/dL			
Visit 1 (enrollment)	11.9 ± 0.5	11.6 ± 0.5	0.3 (−0.1, 0.7)
Visit 2 (late third trimester)	11.7 ± 0.4	11.5 ± 0.4	0.2 (−0.2, 0.5)
Visit 3 (4 mo postpartum)	13.2 ± 0.5	13.1 ± 0.5	0.1 (−0.2, 0.5)
Visit 4 (9 mo postpartum)	12.5 ± 0.5	12.3 ± 0.5	0.2 (−0.2, 0.7)
Difference in difference			0.02 (−0.1, 0.2)
RBP <1.17 μmol/L, *n* (%)			
Visit 1 (enrollment)	57 (22.9)	58 (22.8)	1.0 (0.6, 1.5)
Visit 2 (late third trimester)	73 (41.5)	83 (40.9)	1.0 (0.7, 1.3)
Visit 4 (9 mo postpartum)	26 (13.9)	40 (21.1)	0.6 (0.3, 0.9)*
Difference in difference			0.9 (0.8, 1.1)
Anemia,^5^ *n* (%)			
Visit 1 (enrollment)	80 (32.0)	79 (31.0)	1.0 (0.7, 1.5)
Visit 2 (late third trimester)	66 (37.1)	99 (48.5)	0.5 (0.4, 0.6)^‡^
Visit 3 (4 mo postpartum)	69 (36.1)	88 (43.1)	0.8 (0.6, 1.2)
Visit 4 (9 mo postpartum)	52 (27.7)	51 (26.8)	0.7 (0.3, 1.3)
Difference in difference			1.1 (0.9, 1.2)

1 Values are cluster- and covariate-adjusted least-squares means ± SEs unless otherwise indicated. A total of 505 women (250 INT, 255 CON) were enrolled in early to midpregnancy and followed through 9 mo postpartum and had data available at enrollment; 383 (178 INT, 205 CON) women had data available at visit 2, 401 (196 INT, 205 CON) at visit 3, and 385 (193 INT, 192 CON) at visit 4. *P < 0.05, ^‡^*P* < 0.01. CON, control; INT, intervention; Mama SASHA, Sweetpotato Action for Security and Health in Africa; MUAC, midupper arm circumference; RBP, retinol-binding protein.

2 Estimates are differences and 95% CIs by visit from restricted maximum likelihood estimations by using mixed-effects models.

3 RBP was corrected for inflammation by using the correction factor method described by Thurnham and McCabe (22).

4 Hemoglobin was adjusted for altitude as described by WHO (8).

5 Anemia was defined as altitude-adjusted hemoglobin <110 g/L in pregnancy and 120 g/L in the postpartum period (8).

Mean MUAC was significantly higher among control women at enrollment (*P* < 0.001; Table 5). However, differences between the 2 groups diminished as MUAC tended to decline in the control group over the follow-up period (*P*-trend = 0.14).

## Discussion

### 

Limited research has examined the effects of multisectorial, nutrition-sensitive strategies, such as agriculture, on the health and nutrition of PLW (30–32). Previous OFSP-focused strategies in Uganda and Mozambique improved child VA intakes and status (1, 2) and reduced child diarrhea (33). Maternal VA intakes also increased (2); however, impacts on other maternal health and nutrition indicators were not examined. We observed that the Mama SASHA project, which linked the promotion and distribution of OFSP to health services and enhanced nutrition education for PLW, improved knowledge and increased OFSP and VA intakes. The intervention also reduced the odds of anemia and low RBP but only at the late prenatal and 9-mo postpartum visits, respectively.

Limited overall improvements in RBP from pregnancy through 9 mo postpartum may be attributed to several issues. First, there was a gradual increase in OFSP production and consumption over the course of the study. Studies have noted that interventions targeting dietary change may require extended exposure before significant changes in nutritional status are observed (34–36). Randomized controlled feeding studies with OFSP (35, 37) and pro-VA biofortified maize (38, 39) noted no significant impacts on serum or breast-milk retinol concentrations after 3 wk to 60 d of daily consumption. Mama SASHA provided OFSP vouchers to women during ANC. The OFSP varieties used in this project mature more rapidly than local varieties (∼4 mo) and so could be harvested earlier. However, if women were delayed in attending their first ANC visit or in redeeming and planting their vines, it is plausible that they were unable to harvest and consume OFSP over a sufficient period of time to shift VA status. Thus, targeting women before they become pregnant—for example, newlywed women—may be a more effective strategy to ensure adequate VA intakes before and throughout pregnancy.

Less than 24% of the women showed inflammation-adjusted RBP <1.17 μmol/L at enrollment in pregnancy. Research suggests that the bioavailability of pro-VA and bioconversion efficiency are influenced by VA status (40–42). Traditional cooking methods have limited effects on carotenoid retention (16, 43, 44); however, if pro-VA–rich foods are consumed with inadequate fat or excessive fiber, the efficiency of bioconversion is reduced (40, 44–47). In a population with a relatively low prevalence of insufficiency, the potential combination of insufficient exposure time coupled with traditional diets low in fat and high in fiber may mute the capacity for OFSP to affect indicators of VA status, despite increased VA intakes.

Although the intervention was associated with significant improvements in knowledge and consumption of VA and OFSP and reduced odds of low RBP at 9 mo postpartum, it is important to note that the changes themselves were modest in magnitude. Although knowledge scores increased among intervention women, still <50% of questions were answered accurately. Despite 76% of intervention women reporting OFSP production in the previous year, at the 9-mo postpartum visit only 56% of intervention women reported any consumption of OFSP in the previous 7 d. The limited magnitude of effects may speak to the general difficulties in achieving nutritional social and behavior change (48) as well as to specific project-related limitations, including low uptake of community-based activities such as the PLW’s clubs.

Currently, few interventions are safe and effective for improving VA status of women before, during, and between pregnancies. Industrial fortification strategies may not reach rural or poor populations (49), and community-based fortification initiatives have limited sustainability (50). The high-dose approach used with young children is contraindicated in women of reproductive age due to teratogenicity. Weekly or daily low-dose VA or β-carotene supplementation during pregnancy is recommended for areas in which night blindness is prevalent (51); however, the strategy’s effectiveness depends on functional health systems to sustain and deliver adequate stocks. Animal-source foods, rich in highly bioavailable, preformed VA, are often inaccessible to poor households and women in many low- and middle-income country contexts (52–54). With improved targeting to women before conception and an emphasis on the consumption of OFSP with fat, it is plausible that OFSP could be a safe and effective option for improving the VA intakes of women before, during, and between pregnancies. Because concentrations of VA in breast milk are influenced by maternal intakes, ensuring adequate intakes of VA by mothers throughout the pregnancy and lactation period would benefit infants as well.

A concern with regard to agricultural interventions that target women is the potential for increased work burden (30, 55). Although we did not measure energy expenditures or time allocated, we noted no evidence of an adverse effect on women’s health. Rather, Mama SASHA may have conferred a protective effect on women’s wasting. Over the course of the follow-up period, MUAC decreased to a greater extent among control women than among intervention women. Although energy intakes at 8–10 mo did not differ in the subsample of participants who completed 24-h recalls, it is plausible that the intervention’s explicit linkages with health services may have conferred overall health benefits that manifested in the protection of maternal nutritional status. Similarly, the explicit linkages with health services may explain impacts on anemia in late pregnancy as well.

#### Study strengths and limitations.

The Mama SASHA project design, intervention activities, and evaluation strategy were informed through extensive formative research and participatory impact pathway analysis (56, 57). The impact pathway and subsequent monitoring and evaluation strategy covered all major areas of inputs, actions, and outcomes including the following: knowledge and attitudes; diet, nutrition, and health; seed systems; production practices; and cross-sectorial (horizontal) integration (5). However, the implementation of Mama SASHA faced several challenges. First, funding and logistical constraints limited the number of clusters. Analytical limitations were potentially introduced due to the small number of clusters (i.e., 8 facilities, 23 sublocations) and the additional level of clustering introduced by the repeated measures of the cohort study. However, neither clustering at the facility nor at the sublocation significantly influenced variance in most outcome measures and the inclusion of these cluster levels as random effects worsened the model fit. Thus, we are confident that bias introduced from geographic clustering was minimal for most outcomes.

RBP, as an indicator for VA status, is highly correlated with serum retinol in most contexts, including in Kenya (58); it is also less invasive and cheaper to analyze, making it a useful indicator for assessing program effects (42). The indicator is not without limitations, however. The choice of RBP cutoffs to indicate marginal or deficient VA status is population specific, and concentrations are influenced by a range of factors, most notably the background levels of inflammation, infection, and obesity (22, 42, 59). These similarly influence serum retinol, which is used to validate RBP and to establish RBP cutoffs (42, 60). For financial and logistical reasons we chose to apply cutoffs generated from a 2009 nationally representative population of PLW in Cameroon (23, 24) with similar background rates of infection (Supplemental Tables 5 and 6). We adjusted our models for inflammation and MUAC to account for the potential effects of underlying infection and maternal over- or underweight. Breast-milk retinol is regarded as a sensitive indicator of VA status (61–63). Quantification of retinol and β-carotene in breast-milk samples collected at 4 and 9 mo postpartum is ongoing, and we expect that future analyses of these data will provide additional insights on the impacts of the Mama SASHA program on indicators of women’s VA status.

The cohort study suffered significant losses to follow-up. Missed visits were especially high for the second visit in late pregnancy and attributed to women returning to their maternal home for delivery. These losses occurred despite continued tracking and follow-up by facility-based research assistants and CHWs in both intervention and control groups and an increase in transportation remuneration midway through the study. Although loss to follow-up overall did not differ by treatment group, more intervention women missed the second visit than did control women, potentially biasing late-pregnancy estimates.

Demotivation of CHWs may be one explanation for losses to follow-up. CHWs contributed substantially to the implementation of Mama SASHA program activities, notably community-based mobilization and implementation of mothers’ clubs. In May 2012, when the cohort study started, the government opted to reduce CHW stipends. Qualitative operations research and monitoring data revealed subsequent declines in CHW engagement in a range of program activities after these cuts, including support for voucher distribution and redemption, mobilization of women, home visits, and implementation of mothers’ clubs. Our team developed nonfinancial incentives to counteract this demotivation, including badges and certificates; these efforts facilitated re-engagement of CHWs but not to the same levels as before the stipend cuts. Future longitudinal studies in pregnant women in rural Kenya may benefit from anticipating larger losses to follow-up in sample size estimates and putting greater emphasis on strategies to retain women and maintain motivation of frontline workers who work with them.

#### Conclusions.

Our findings, when viewed collectively with existing evidence (1–3, 35), support the promotion of OFSP as a viable nutrition-sensitive agriculture strategy that improves nutrition knowledge, the consumption of VA-rich foods, and VA intakes among PLW. Over the long term this strategy may safely and effectively enhance maternal diets and nutrition. Integrating promotion with health services is feasible and may have added nutrition and health benefits; however, additional research is needed to identify cost-efficient strategies that motivate increased participation in community-based nutrition education activities. Furthermore, given the time required to plant and harvest OFSP, even rapidly maturing varieties, it may be beneficial to target women before pregnancy to maximize VA intakes.

## Footnotes

8 Abbreviations used: AGP, α1-acid glycoprotein; ANC, antenatal care; APHIAplus, AIDS, Population, and Health Integrated Assistance Zone 1; CHW, community health worker; CRP, C-reactive protein; DiD, difference in difference; IYCF, infant and young child feeding; Mama SASHA, Sweetpotato Action for Security and Health in Africa; MUAC, midupper arm circumference; OFSP, orange-fleshed sweet potato; PLW, pregnant and lactating women; PNC, postnatal care; RAE, retinol activity equivalent; RBP, retinol-binding protein; VA, vitamin A; WDDS, women’s dietary diversity score.
